# Nano- and Macroscale Study of the Lubrication of Titania Using Pure and Diluted Ionic Liquids

**DOI:** 10.3389/fchem.2019.00287

**Published:** 2019-04-26

**Authors:** Peter K. Cooper, Joe Staddon, Songwei Zhang, Zachary M. Aman, Rob Atkin, Hua Li

**Affiliations:** ^1^School of Molecular Sciences, University of Western Australia, Crawley, WA, Australia; ^2^State Key Laboratory of Solid Lubrication, Lanzhou Institute of Chemical Physics, Chinese Academy of Sciences, Lanzhou, China; ^3^Fluid Science and Resources, Department of Chemical Engineering, University of Western Australia, Crawley, WA, Australia

**Keywords:** titanium, lubrication, friction mechanism, atomic force microscopy, nanotribology, light-weight metal

## Abstract

Titanium is a strong, corrosion-resistant light—weight metal which is poised to replace steel in automobiles, aircraft, and watercraft. However, the titanium oxide (titania) layer that forms on the surface of titanium in air is notoriously difficult to lubricate with conventional lubricants, which restricts its use in moving parts such as bearings. Ionic liquids (ILs) are potentially excellent lubricants for titania but the relationship between IL molecular structure and lubricity for titania remains poorly understood. Here, three-ball-on-disk macrotribology and atomic force microscopy (AFM) nanotribology measurements reveal the lubricity of four IL lubricants: trioctyl(2-ethylhexyl)phosphonium bis(2-ethylhexyl)phosphate (P_8,8,8,6(2)_ BEHP), trihexyl(tetradecyl)phosphonium bis(2-ethylhexyl)phosphate (P_6,6,6,14_ BEHP), trihexyl(tetradecyl)phosphonium bis(2,4,4-trimethylpentyl)phosphinate (P_6,6,6,14_ (^*i*^C_8_)_2_PO_2_), and trihexyl(tetradecyl)phosphonium bis(trifluoromethylsulfonyl)imide (P_6,6,6,14_ TFSI). The macrotribology measurements demonstrated that friction decreased in P_6,6,6,14_ TFSI by four times (μ = 0.13) compared to in hexadecane, even at 60°C and loads up to 10 N. On the other hand, P_8,8,8,6(2)_ BEHP reduced friction most effectively in the AFM nanotribology measurements. The results were interpreted in terms of the lubrication regime. The lower viscosity of P_6,6,6,14_ TFSI coupled with its good boundary lubrication made it the most effective IL for the macrotribology measurements, which were in the mixed lubrication regime. Conversely, the cation structure of P_8,8,8,6(2)_ BEHP allowed it to adsorb strongly to the surface and minimized energy dissipation in the nanotribology measurements, although its high bulk viscosity inhibited its performance in the mixed regime. These results reinforce the importance of carefully selecting IL lubricants based on the lubrication regime of the sliding surfaces.

## Introduction

Recent interest in optimizing efficiency in machines has led to a strong desire to replace steel with light—weight materials. Titanium is an attractive candidate, with the highest strength-to-density ratio of any metallic element, and excellent resistance to corrosion and heat (Budinski, 1991). These properties make titanium an exceptional material for stationary parts in machinery and are why it is increasingly adopted in automobiles, aircraft, and watercraft (Schutz and Scaturro, 1991; Boyer, 1996; Faller and Froes, 2001).

A critical drawback of titanium is its poor lubrication. Conventional lubricant additives such as zinc dialkyldithiophosphate (ZDDP) do not lubricate titanium well, which restricts its use in moving parts such as bearings (Rabinowicz and Kingsbury, 1955; Tian et al., 1989). Furthermore, titanium tends to strongly adhere to other surfaces, resulting in adhesive wear known as galling (Budinski, 1991; Dong, 2010). Presently, the best anti-wear protection comes from oxidizing the surface under very high temperatures (>800°C) to form a corrosion resistant titanium dioxide (titania) film (Qu et al., 2009; Bailey and Sun, 2013). Apart from being expensive, this treatment is potentially ineffective over long periods of time (Bansal et al., 2013). A more desirable solution involves using a liquid lubricant which can reduce friction and wear, as well as dispersing wear particles and dissipating heat generated from friction.

Ionic liquids (ILs) are potentially excellent lubricants for titania. ILs are pure salts that are liquid at low temperatures. Their exceptional physical properties including high thermal stability and negligible vapor pressures mean that they can be used in high temperature or low pressure environments such as outer space (Kobayashi et al., 2015; Nancarrow and Mohammed, 2017). Furthermore, ILs interact strongly with themselves and surfaces via electrostatic, van der Waals, h-bonding and solvophobic interactions (Jiang et al., 2018). This rich range of interactions enables them to act as highly effective boundary lubricants, where both the cation and anion adsorb strongly to a wide variety of surfaces and form a protective lubricating film. Both nano- and macroscale friction and wear measurements have demonstrated that ILs effectively lubricate steel, alumina, silica and diamond—like carbon (Somers et al., 2013; Qu et al., 2015; Li et al., 2016a; Cooper et al., 2017; Cowie et al., 2017; Zhou and Qu, 2017).

In contrast to the extensive studies of ILs lubricating other surfaces, very few studies have been carried out into IL lubrication for titanium surfaces (Jiménez and Bermúdez, 2009, 2010a,b). Li et al. showed that the IL trihexyl(tetradecyl)phosphonium bis(2,4,4-trimethyl)pentylphosphinate (P_6,6,6,14_ (^*i*^C_8_)_2_PO_2_) reduced the friction coefficient five times more effectively than a base oil without additives (Li et al., 2016b). Importantly, diluting the IL in a hydrocarbon oil above 2 mol% showed reduced friction almost to the same extent as the pure IL.

While some ILs have been shown to be good lubricants for titania, the relationship between IL structure and lubrication remains only partially understood. Under low loads, ILs, like conventional lubricants, protect sliding surfaces by physically separating them via hydrodynamic pressure. This is known as hydrodynamic lubrication. If the viscosity of the lubricant is too high, however, hydrodynamic drag leads to high friction. At higher loads, the surfaces intermittently come into direct contact with each other, and friction results from a combination of hydrodynamic friction and energy lost through the surface contact. This is referred to as mixed or elastohydrodynamic lubrication. At very high loads, the liquid separating the surfaces is squeezed out, and lubrication comes from a molecularly thin layer of lubricant between the surface, and is known as boundary lubrication.

In this study, macroscale three-balls-on-disk and nanoscale AFM friction force microscopy (FFM) tribology measurements reveal that the lubricity of the IL depends strongly on the bulk viscosity as well as the IL structure. Whereas the macroscale experiment measures the average friction over a relatively large area, AFM FFM measures the friction between single asperities on the surfaces (Enachescu et al., 1998; Enachescu, 2012). Four quaternary phosphonium ILs with subtle differences in their structure were selected. Quaternary phosphonium—based ILs have attracted considerable attention as lubricants in recent years as they can dissolve in apolar hydrocarbon oils including mineral oil (Yu et al., 2012; Zhou and Qu, 2017). As an additional benefit, quaternary phosphonium—based ILs are generally very stable, hydrophobic and do not lead to severe corrosion observed in other IL lubricants (Somers et al., 2013; Zhou and Qu, 2017). In addition to the measurements of the pure ILs, measurements of the ILs diluted in a model hydrocarbon oil were also carried out to investigate their effectiveness as additives.

## Materials and Methods

### Ionic Liquids

The ILs trihexyl(tetradecyl)phosphonium bis(2-ethylhexyl)phosphate (P_6,6,6,14_ BEHP) (purity >98 %), trihexyl(tetradecyl)phosphonium bis(2,4,4-trimethylpentyl)phosphinate (henceforth referred to as P_6,6,6,14_ (^*i*^C_8_)_2_PO_2_) (purity >95 %), and trihexyl(tetradecyl)-phosphonium bis(trifluoromethylsulfonyl)imide (henceforth referred to as P_6,6,6,14_ TFSI) (purity >98 %) were all supplied by IoLiTec and used as received. Trioctyl(2-ethylhexyl)phosphonium bis(2-ethylhexyl)phosphate (P_8,8,8,6(2)_ BEHP) was synthesized by adding an equimolar amount of trioctylphosphine dropwise to trioctylphosphate. The reaction was carried out under nitrogen at a temperature between 180 and 200°C over 30 h. The product was purified through high pressure distillation, with a yield of ~75% and viscosity measured to be ~1,050 mPa · s. The viscosity and molecular structure of all the ILs are provided in Table 1. Hexadecane (purity >99%), purchased from Sigma Aldrich was used as a hydrocarbon oil with which the friction coefficients using the ILs could be compared to.

**Table 1:** The viscosities and molecular structures of the ILs used in this study.

**Ionic liquid**	**Viscosity (mPa s)**	**Molecular structure**
P_8,8,8,6(2)_ BEHP	1050	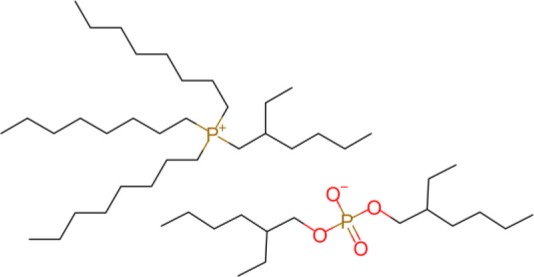
P_6,6,6,14_ BEHP	1045^a^ (Barnhill et al., 2014)	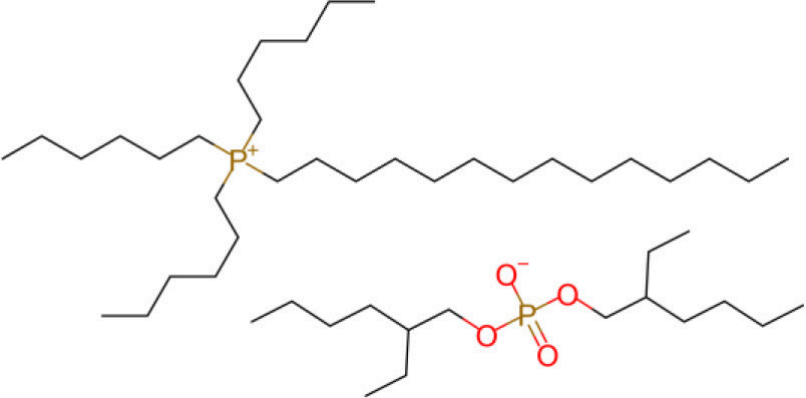
P_6,6,6,14_ (*^*i*^*C_8_)_2_PO_2_	1007^b^ (Li et al., 2016a)	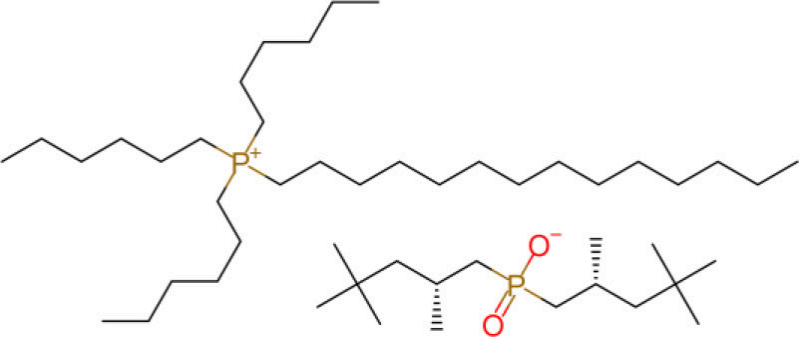
P_6,6,6,14_ TFSI	312^b^ (Fraser et al., 2007)	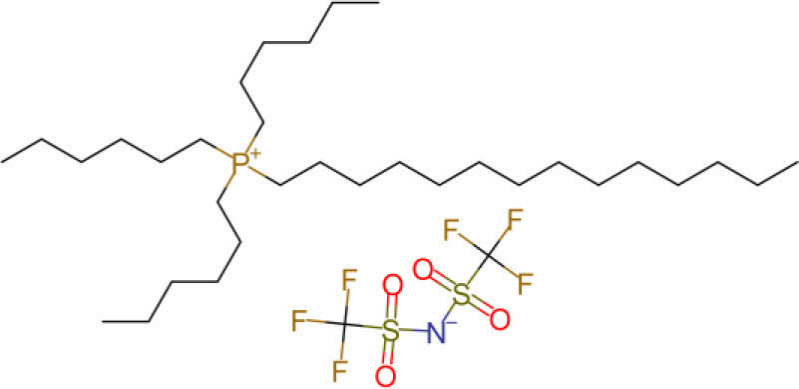

a *Measured at 23°C*.

b *Measured at 25°C*.

### Titanium Surface

The titanium surfaces used in the experiments were cut from a single sheet of unpolished titanium metal (Airport Metals Australia). After initial investigations on a polished titanium surface, it was decided that an unpolished, commercially available titanium surface should be used. This is a more industrially relevant surface for lubrication, and thus more suitable for macroscale friction measurements. The RMS roughness for a 5 × 5 μm scan of the titanium surface was measured to be 108 ± 16 nm by AFM.

### Macrotribology

Macroscale friction measurements were taken using a TA Instruments HR-2 rheometer with a three-balls on plate geometry using stainless-steel balls (diameter = 12.7 mm) on the titanium surface. The sliding speed was a constant 15 mm s^−1^, and the motor mode set to “Stiff.” Varying environmental conditions were used, with the temperature of the base set to 25 and 60°C, and the axial force set to 5 and 10 N. The coefficient of friction, μ, was recorded as the ratio between the lateral and normal force. Each condition was recorded for 9 m of sliding. The test in P_6,6,6,14_ TFSI at 5 and 10 N was recorded for 25 m to investigate the strength of the boundary layer. The titanium surfaces were cleaned with ethanol and air dried before reuse. The friction coefficient was measured in air after cleaning to ensure that it was consistent with the friction coefficient of the fresh surface before the sliding tests. The ILs were used on different surfaces to prevent contamination.

### Atomic Force Microscopy

A Veeco Nanoscope IV AFM with an EV scanner was used in contact mode. Sharp silicon tips with a spring constant of 0.8 ± 0.2 N m^−1^ as measured by the Sader method (Sader et al., 1999) and a nominal tip radius of 8 nm (HQ:NSC36/ AL BS, Mikromasch) were used in these experiments. The measurements were performed inside an AFM fluid cell (Bruker). Before each experiment the tip was irradiated with ultraviolet light for 10 min to remove any organic matter, and the surface and cell washed with ethanol and deionized water before being dried with nitrogen.

Friction measurements were performed with a scan angle of 90° (with respect to the cantilever long axis), a scan size of 500 nm and a scan rate of 5.92 Hz with the slow scan axis disabled while the normal load was increased from 0 to 200 nN. The lateral deflection signal (i.e., cantilever twist) was converted to lateral force using a customized function produced in MATLAB. This function converts the lateral voltage trace and retrace data into friction force by taking into account the torsional spring constant, and the lateral and normal deflection sensitivity. More details are provided in Pettersson and Dédinait (2008). The lateral deflection sensitivity was calculated by the method described by Schwarz et al. (1996). The friction coefficient, μ, of each data set was extracted from the linear region of the lateral force vs. normal load graph, in accordance with Amontons' Law, *F*_L_ = μ*F*_N_ + *F*_L_(0), where *F*_L_(0) is the lateral force at zero normal load (Enachescu et al., 1999). At least three runs of increasing and decreasing load were performed for each liquid, and each run performed on a different area of the titanium. Increasing and decreasing load showed the same trend, so the decreasing load data have been omitted for clarity.

Force separation profiles were recorded for each liquid by moving the surface toward the tip and detecting the cantilever deflection as a function of separation, with at least 60 normal force curves recorded from a ramp size of 30 nm, at a scan rate of 0.2 Hz. Standard methods were used to convert deflection vs. separation data to normal force vs. apparent separation curves.

## Results and Discussion

### Macrotribology

The macrotribology of the ILs P_6,6,6,14_ BEHP, P_8,8,8,6(2)_ BEHP, P_6,6,6,14_ (^*i*^C_8_)_2_PO_2_ and P_6,6,6,14_ TFSI was studied with a three-balls on disk tribometer. The coefficient of friction was measured over 9 m for each IL at 5 and 10 N of load at both 25 and 60°C. The balls were spun at a speed of 15 mm s^−1^. 5 N load corresponds to a Hertzian contact stress of 0.5 GPa for each ball, while 10 N corresponds to 0.6 GPa (see Supplementary Materials for calculations). For comparison, the same tests were performed with hexadecane as a model, additive-free hydrocarbon oil.

**Figure 1:**
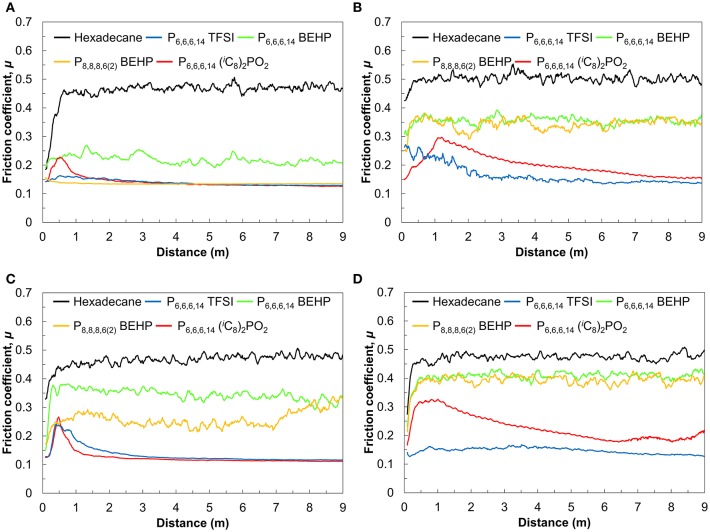
Friction coefficient as a function of sliding distance at **(A)** 25°C and 5 N, **(B)** 25°C and 10 N, **(C)** 60°C, and 5 N, **(D)** 60°C and 10 N.

The friction coefficient vs. sliding distance measured in the ILs and hexadecane is shown in Figure 1. Over the first 1 m, the friction coefficient varies significantly before reaching a stable value. This initial period of instability is referred to as the “break-in” period (Neale, 1995). In hexadecane, the friction coefficient increases rapidly as the unprotected surfaces are quickly worn down before it stabilizes at ~0.5 regardless of the load or temperature. This is consistent with the friction coefficient of titania in hexadecane measured in previous work (Qu et al., 2005; Li et al., 2016b). Hexadecane still lubricates the surfaces better than in air (c.f. Figure S1), where friction continues to increase with sliding distance since the wear debris is not transported away from the sliding zone as it is in a liquid.

Under a load of 5 N at 25°C (Figure 1A), the friction coefficient decreased up to 2–5 times in ILs compared to the friction coefficient in hexadecane. P_6,6,6,14_ TFSI and P_8,8,8,6(2)_ BEHP reduced the friction coefficient to 0.13 almost immediately without any significant break-in period. On the other hand, in P_6,6,6,14_ (^*i*^C_8_)_2_PO_2_, friction initially increased, then decreased, with low friction (μ = 0.13) only observed after 2 m of sliding. Of the four ILs tested, P_6,6,6,14_ BEHP was the least effective (μ = 0.22), albeit still much more effective than hexadecane.

To investigate the effectiveness of the ILs under more demanding conditions, the same measurements were performed under a higher load of 10 N and the temperature increased from 25 to 60°C. Under 10 N at 25°C (Figure 1B), the ILs P_6,6,6,14_ TFSI and P_6,6,6,14_ (^*i*^C_8_)_2_PO_2_ reduced friction as effectively as they did at 5 N (μ = 0.13), albeit the break-in period increased significantly from that at 5 N. Conversely, the friction coefficient for the ILs P_8,8,8,6(2)_ BEHP at 10 N was much higher than at 5 N (μ = 0.28 vs. 0.13). Similarly, P_6,6,6,14_ BEHP demonstrated a higher friction coefficient under 10 N of load than 5 N (μ = 0.35 vs. 0.22).

The tests at 60°C also revealed differences in the performance of the ILs which were not observed at 25°C. At 60°C and 5 N (Figure 1C), P_6,6,6,14_ TFSI lubricates as well as at 25°C (μ = 0.13), albeit only after 2 m of sliding. The friction coefficient in P_6,6,6,14_ (^*i*^C_8_)_2_PO_2_ varied, from an initial increase to 0.30 over the first 2 m, followed by a gradual decrease in friction coefficient, eventually reaching 0.15 after 9 m. P_6,6,6,14_ BEHP and P_8,8,8,6(2)_ BEHP were less effective at reducing friction, with μ = 0.35 in both cases. At 10 N of load (Figure 1D), the trends were similar to 5 N, only differing in a slightly higher friction coefficient for P_6,6,6,14_ (^*i*^C_8_)_2_PO_2_, P_6,6,6,14_ BEHP and P_8,8,8,6(2)_ BEHP.

**Figure 2:**
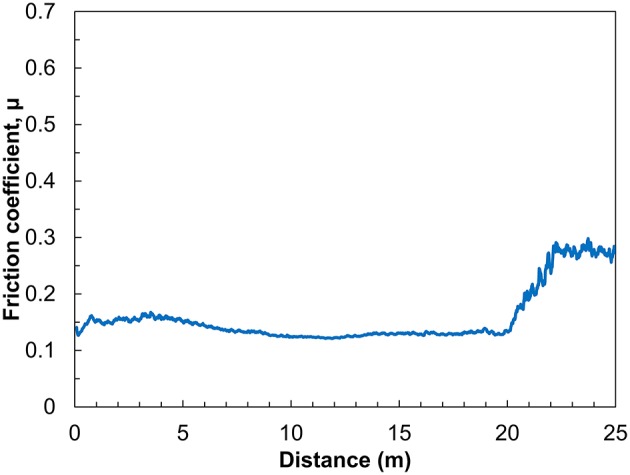
Friction coefficient as a function of sliding distance for three stainless steel balls sliding on a titanium surface in P_6,6,6,14_ TFSI. Sliding was carried out under 10 N of normal load and a temperature of 60°C.

The measurements at higher loads and temperatures demonstrate that P_6,6,6,14_ TFSI lubrication reduces friction more effectively than any of the other ILs. Longer tests reveal the durability of P_6,6,6,14_ TFSI lubricity at 60°C and 10 N and are presented in Figure 2. Between 2 and 20 m of sliding, the friction coefficient in P_6,6,6,14_ TFSI is low and stable. However, after 20 m of sliding the friction coefficient increases until it seems to stabilize at 0.25. The breakdown in lubrication has been observed previous for IL macrotribology (Li et al., 2016a). The sudden increase in friction suggests that the ball suddenly ruptures the boundary layer, which is subsequently unable to reform. Even still, after the breakdown the friction coefficient in lubrication is half of that measured in hexadecane. This indicates that lubrication between the surfaces persists, albeit perhaps only via hydrodynamic lubrication.

Mixtures of the ILs with hexadecane did not reduce friction as effectively as the pure ILs. 1 mol% mixtures of P_6,6,6,14_ (^*i*^C_8_)_2_PO_2_, P_8,8,8,6(2)_ BEHP and P_6,6,6,14_ BEHP with hexadecane were measured at 25°C under 5 N of load and are presented in Figure S2. P_6,6,6,14_ TFSI is immiscible with hexadecane and therefore could not be used. The mixtures did not reduce friction more effectively than hexadecane. Measurements of the mixtures at 60°C and 10 N did not show any signs of improvement over hexadecane.

While the results demonstrate that P_6,6,6,14_ TFSI lubricates most effectively under all the tested conditions, it is not immediately obvious why. The thickness of the IL films was estimated to be between 18 and 74 nm by the Hamrock and Dowson model, as shown in Table S1 (Stachowiak and Batchelor, 2005), which suggests that the ILs are in a mixed lubrication regime (calculations provided in the Supplementary Material). Additionally, measuring *μ* vs. velocity for P_6,6,6,14_ TFSI and hexadecane (c.f. Figure S3) reveals that the experiments were carried out at the “trough” of the Stribeck curve i.e., in the mixed region (Stachowiak and Batchelor, 2005). In the mixed lubrication regime, friction results from a combination of intermittent direct contact between the surfaces as well as hydrodynamic losses. However, the trends in friction coefficient data do not correlate with the trends in viscosity provided in Table 1. This means that the ILs lubricate the direct contact between the surfaces differently via boundary lubrication, and that it is not purely hydrodynamic losses via viscosity.

### Nanotribology

AFM friction force microscopy measurements were performed to disentangle the contribution from boundary layer lubrication and that of mixed layer lubrication in the macrotribology measurements. A sharp Si tip was used with the same type of titanium surface used in the macrotribology. The lateral (friction) force was recorded up to 200 nN. A 200 nN load equals a Hertzian contact stress of 18.7 GPa, assuming that the tip dimensions are not significantly altered during the friction experiments. These forces correspond to the boundary regime; calculation of the film thickness revealed an impossibly thin film (<1 pm), consistent with previous AFM boundary layer studies (Li et al., 2016a,b). In the boundary regime, the bulk of the liquid lubricant has been squeezed out, and lubrication is effected by a layer of molecules adsorbed at the surface, called the boundary layer. Boundary lubrication is therefore independent of the bulk viscosity of the liquid.

**Figure 3:**
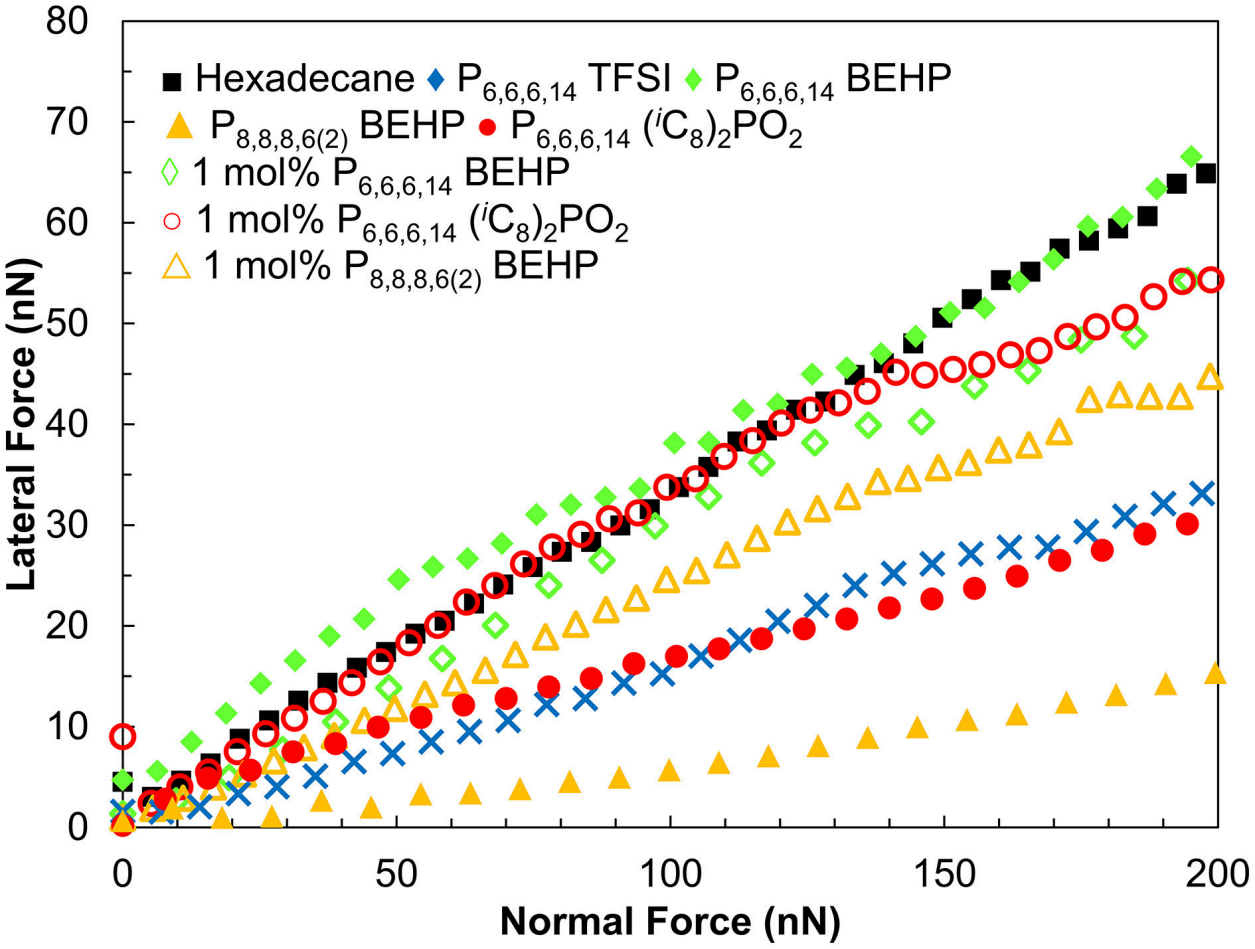
Lateral (friction) force vs. normal force for a sharp Si AFM tip sliding over a titanium surface at 35 μm s^−1^.

The lateral forces experienced by the tip as it slides on the surface as a function of normal load are shown in Figure 3. The gradient of these data is the friction coefficient μ as per Amontons' law *F*_*L*_ = *F*_*A*_ + μ*F*_*N*_, where *F*_*L*_ is the lateral force, *F*_*A*_ is the force of adhesion, and *F*_*N*_ is the normal load. In hexadecane, the friction force increases rapidly with increasing normal load, with μ = 0.33. This reveals that hexadecane is, as expected, a poor lubricant. As the tip and the surface come into contact any weakly adsorbed hexadecane is expelled resulting in direct contact between the tip and surface and high friction.

In contrast to hexadecane, P_8,8,8,6(2)_ BEHP is an excellent boundary lubricant. Lateral forces increase at a lower rate with increasing normal force, resulting in a friction coefficient of 0.08, approximately four times lower than in hexadecane. P_6,6,6,14_ (^i^C_8_)_2_PO_2_ and P_6,6,6,14_ TFSI, the most effective ILs at reducing friction on the macroscale, reduced friction by about half of that measured in hexadecane (μ = 0.16 and 0.17 respectively). P_6,6,6,14_ BEHP did not reduce friction any more effectively than hexadecane (μ = 0.33), which is consistent with it being the poorest lubricant on the macroscale. A summary of the friction coefficients measured by AFM are presented in Table 2.

**Table 2:** The calculated friction coefficients of the ILs measured by AFM.

**Liquid**	**Friction coefficient, μ**
P_8,8,8,6(2)_ BEHP	0.08
P_6,6,6,14_ BEHP	0.33
P_6,6,6,14_ (^i^C_8_)_2_PO_2_	0.16
P_6,6,6,14_ TFSI	0.17

As with the macroscale tests, 1 mol % mixtures of P_8,8,8,6(2)_ BEHP, P_6,6,6,14_ (^*i*^C_8_)_2_PO_2_ and P_6,6,6,14_ BEHP in hexadecane did not significantly reduce friction (Figure 3). Previous studies of IL/oil mixtures on surfaces including titania have shown that 1–2 mol% mixtures can reduce the friction coefficient as effectively as the pure IL (Li et al., 2014, 2016b; Cooper et al., 2016). However, these reductions in friction only occur after the bulk concentration reaches a critical concentration (Li et al., 2014). Surfactant adsorption studies with titania have found that the surfactant surface excess may be half of that compared to another oxide surface such as silica (Favoriti et al., 1996). A lower surface excess means that the adsorbed boundary layer of IL will be patchy, and unable to reduce friction as effectively.

The trends in the AFM nanotribology results presented in Figure 3 can be explained by considering the mechanisms in which energy dissipation (i.e., friction) occurs. In the boundary regime, energy dissipates mainly via (i) expulsion of the near surface IL layers from the space between the tip and the surface, and (ii) by deformations and rotations of ions in the boundary layer (Salmeron, 2001; Sweeney et al., 2014). A common feature of many ILs is the presence of layers of adsorbed ions at solid surfaces which are preferentially oriented based on the polar and apolar domains of the IL. Number of observed layers varies significantly with IL molecular structure. Previous AFM nanotribology measurements have demonstrated that the presence of these layers in ILs is inversely proportional to friction; that is, the greater the number of near surface layers, the more energy dissipates via (i) (Sweeney et al., 2014; Cooper et al., 2017).

**Figure 4:**
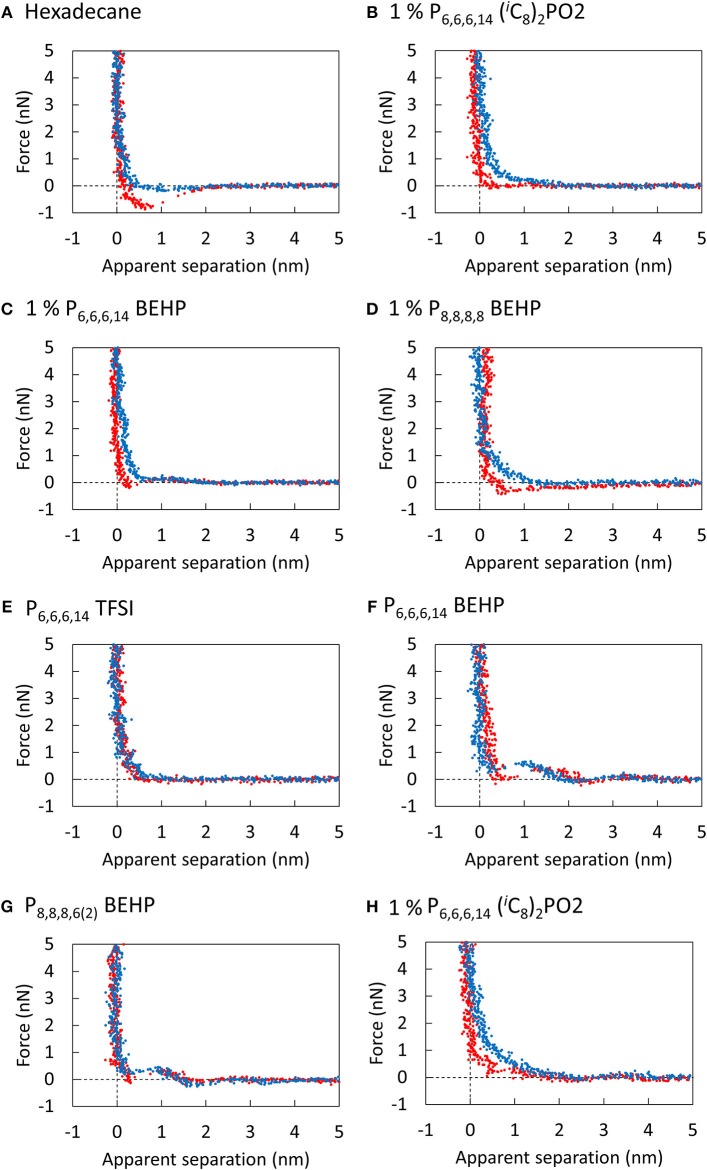
Force curves of the silicon AFM tip approaching (blue) and retracting (red) from the unpolished titanium surface submerged in **(A)** Hexadecane, **(B)** 1% P_6,6,6,14_ (^*i*^C_8_)_2_PO_2_, **(C)** 1% P_6,6,6,14_ BEHP, **(D)** 1% P_8,8,8,6(2)_ BEHP, **(E)** P_6,6,6,14_ TFSI, **(F)** P_6,6,6,14_ BEHP, **(G)** P_8,8,8,6(2)_ BEHP, and **(H)** 1% P_6,6,6,14_ (^*i*^C_8_)_2_PO_2_. Scan size: 30 nm.

To investigate how significant the near surface structure is in these ILs, a series of AFM normal force—separation profiles were measured for the pure ILs and the mixtures and are presented in Figure 4. For each IL and mixture, no significant force was measured beyond 2 nm of apparent separation between the tip and the surface. The “steps” in the force profile at closer separations indicate the presence of a single near surface layer in some cases, however, all ILs in this study are generally weakly unstructured. Based on the weak structure observed by AFM, energy dissipation via (i) cannot explain the large differences in lateral forces measured in the ILs.

The differences in the lateral forces measured by nanotribology may instead be explained by considering how the ions adsorb and pack onto the surface, which determines energy dissipation via (ii). The isoelectric point of titania is 5.7, and therefore one might expect the cation to preferentially adsorb to the surface. Nevertheless, neutron reflectometry measurements and MD simulations have revealed that oppositely charged ions still contribute to the adsorbed layer (Lauw et al., 2012; Wang et al., 2017; Cooper et al., 2018).

Considering the cations first, Figure 2 shows that P_8,8,8,6(2)_ BEHP reduces friction far more effectively than P_6,6,6,14_ BEHP. The P_8,8,8,6(2)_+ cation can pack more neatly with the bulky BEHP^−^ ion than ${\text{P}}_{6,6,6,14}^{+}$ with its long 14 C chain potentially sterically hindering one of the ions or both from adsorbing strongly on the surface. For the anions, comparing P_6,6,6,14_ BEHP, P_6,6,6,14_ (^*i*^C_8_)_2_PO_2_ and P_6,6,6,14_ TFSI, the TFSI^−^ and (^*i*^C_8_)_2_${\text{PO}}_{2}^{-}$ were approximately equally effective. This result is consistent with the results from a previous study on stainless steel (Cooper et al., 2017). The BEHP^−^ anion in this case was the worst performing of the three. Despite having an apparently similar structure to (^*i*^C_8_)_2_${\text{PO}}_{2}^{-}$, the longer alkyl chains of the BEHP^−^ restrict its packing compared to the more dense (^*i*^C_8_)_2_${\text{PO}}_{2}^{-}$ and TFSI^−^ anions. Indeed, MAS NMR, FITR and Raman spectroscopy measurements have shown that the interaction between the anions of quaternary phosphonium ILs and metal oxide surfaces can vary significantly, despite similar molecular structures (Shah et al., 2018).

## Conclusions

Combining the results from the macroscale tribology experiments and the nanotribology experiments gives remarkable insight into the role of IL structure on friction across both length scales. All ILs in this study were generally good lubricants for titania. However, IL lubrication performance in the nanotribology tests varied from the macrotribology tests. For the AFM nanotribology, only the structure of the ILs determines boundary lubrication. More specifically, the ability of the ions to pack into a neat, robust layer largely determined their success as nanoscale lubricants. On the other hand, for the macroscale three-ball-on-disk experiments, a combination of bulk physical properties and adsorbed boundary layer govern mixed layer lubrication. This difference is illustrated by P_8,8,8,6(2)_ BEHP, which was the most effective IL in the nanotribology tests, but only the third most effective in the macrotribology tests.

For lubrication in the boundary regime lubrication, the ideal IL will adsorb strongly to the surface and form a robust film which minimizes energy dissipation. Importantly, for boundary regime lubrication the viscosity of the IL does not need to be considered. For lubrication in the mixed or hydrodynamic regime, the macrotribology measurements here show the importance of both viscosity and boundary layer lubrication. Ultimately, this work demonstrates the importance of the judicious selection of IL based on the lubrication regime operating in the sliding surfaces.

## Author Contributions

HL, RA, and ZA designed the experiments, SZ synthesized P_8,8,8,6(2)_ BEHP, and JS and HL performed the experiments. PC, JS, and HL analyzed the data and PC and RA wrote the manuscript. The manuscript was written through the substantial contributions of all authors.

### Conflict of Interest Statement

The authors declare that the research was conducted in the absence of any commercial or financial relationships that could be construed as a potential conflict of interest.

## Supplementary Material

Data Sheet 1 These include the macrotribology friction coefficient vs. sliding distance measured in air, the friction coefficient vs. sliding distance measured in the 1 mol% mixtures of IL in hexadecane, the AFM force—separation curves, and the AFM lateral force vs. normal force data for the 1 mol% mixtures of the ILs.
