# Supplementary material for: Nano- and Macroscale Study of the Lubrication of Titania Using Pure and Diluted Ionic Liquids
Source: Front Chem. 2019 Apr 26;7:287. doi: 10.3389/fchem.2019.00287 (PMC6498968; doi:10.3389/fchem.2019.00287)
Supplement: Data Sheet 1 — These include the macrotribology friction coefficient vs. sliding distance measured in air, the friction coefficient vs. sliding distance measured in the 1 mol% mixtures of IL in hexadecane, the AFM force—separation curves, and the AFM lateral force vs. normal force data for the 1 mol% mixtures of the ILs. [file Data_Sheet_1.pdf]

Supplementary Material
Nano- and macroscale study of the lubrication of titania using pure and diluted ionic liquids

Peter K. Cooper,[a,#] *Joe Staddon,*[a,#]*, Songwei Zhang,*[b] *Zachary M. Aman,*[c] *Rob Atkin*[a,*]

and Hua Li,[a,*]

[a] School of Molecular Sciences, University of Western Australia, Crawley, Western Australia, Australia.

[b] State Key Laboratory of Solid Lubrication, Lanzhou Institute of Chemical Physics, Chinese Academy of Sciences, Lanzhou, P. R. China.

[c] Fluid Science and Resources, Department of Chemical Engineering, University of Western Australia, Crawley WA, Australia

#These authors contributed equally.

*Corresponding authors emails: hua.li@uwa.edu.au, rob.atkin@uwa.edu.au

Macrotribology

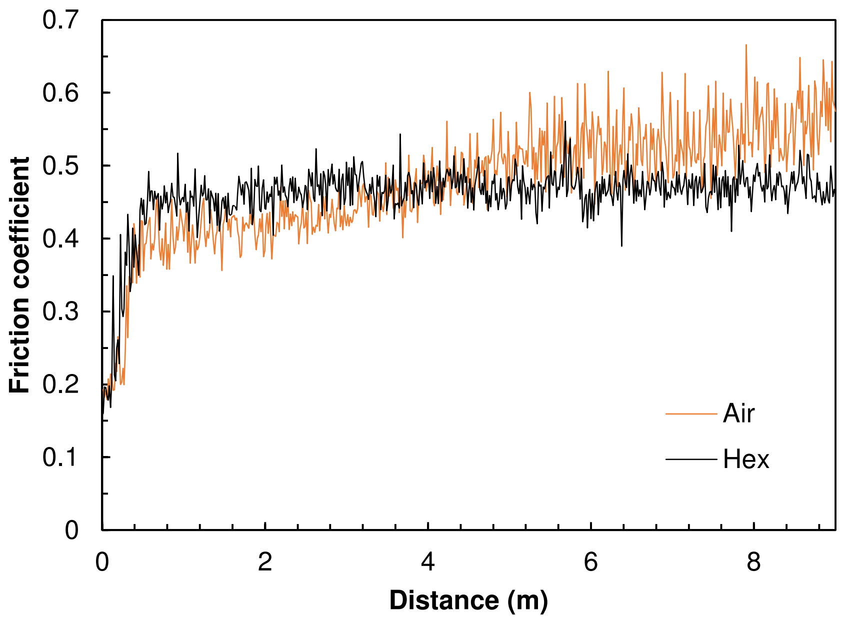

FIGURE S1 | Friction coefficient vs sliding distance measured in air and hexadecane under a load of 5 N and at 25 ℃.

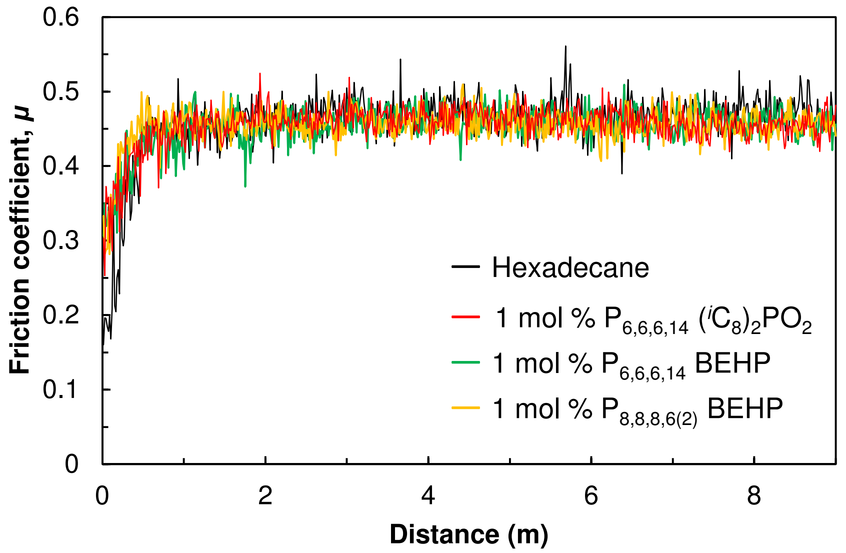

FIGURE S2 | Friction coefficient as a function of sliding distance for three stainless steel balls sliding on a titanium surface in 1 mol % mixtures of IL in hexadecane. Sliding was carried out under 5 N of normal load and a temperature of 25 ℃.

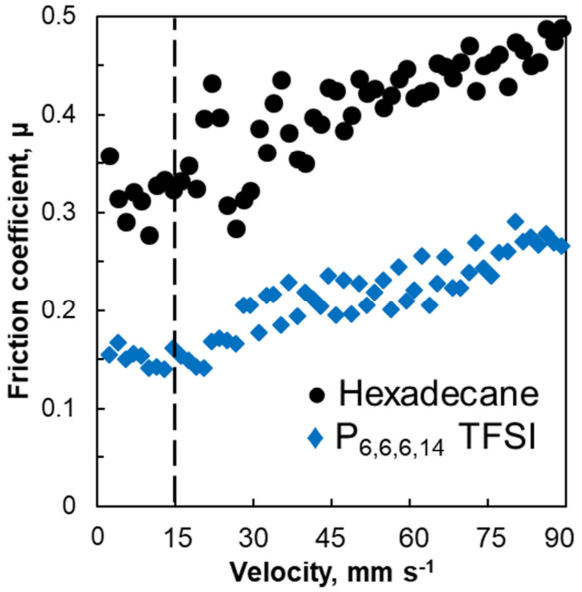

FIGURE S3 | Friction coefficient as a function of velocity for three stainless steel balls sliding on a titanium surface in hexadecane (black circles) and $P_{6,6,6,14}$ TFSI (blue diamonds). The dashed line shows the velocity at which the other experiments were carried out. Sliding was carried out under 5 N of normal load and a temperature of 60 ℃.

Calculation of Hertzian Contact Stress

The effective Young's modulus E_w can be calculated by:

$$\frac{1}{E_w} = \frac{1}{2}\left(\frac{1-v_1^2}{E_1} + \frac{1-v_2^2}{E_2}\right)$$

Where E_1, E_2 are the elastic moduli and v_1, v_2 are the Poisson's ratios associated with each body.

Contact area radius is given by:

$$a \cong \left(\frac{3RF}{2E_w}\right)^{1/3}$$

Where R is the radius of the sphere. F is the applied load.

Contact stress:

$$p_{max} = \frac{3F}{2\pi a^2}$$

The parameters used:

Titania: E = 230 GPa, v = 0.28

Stainless Steel: E = 200 GPa, v = 0.3

Macrotribology test:

F = 5 N, $p_{max} = 0.5\ GPa$

F = 10 nN, p_{max} = 0.6 GPa

In the nanotribology tests the exact dimensions and geometry of the tip are uncertain and subject to change. The contact pressures are significantly higher:

F = 50 nN, p_{max} = 11.8 GPa

F = 200 nN, p_{max} = 18.7 GPa

Hamrock and Dowson model

Using the Hamrock and Dowson model the central film thickness, h_c, can be calculated by the following equation (Stachowiak and Batchelor, 2005):

$$\frac{h_c}{R'} = 2.69\left(\frac{U\eta_0}{E'R'}\right)^{0.67}(\alpha E')^{0.53}\left(\frac{W}{E'R'^2}\right)^{-0.067}(1-0.61e^{-0.73k})$$

where a is the contact area radius from eq(2), U is the sliding velocity, η_0 is the viscosity of the lubricant at ambient pressure, E_w is the effect Young's modulus from eq(1), α is the pressure-viscosity coefficient, F is the applied load, and k is the ellipticity parameter. k = 1 for point contact. The pressure – viscosity coefficient of hexadecane varies from 11.6 to 13.2, and average of 12.5 is used here (Pensado et al., 2008; Paredes et al., 2012). For the pure ILs used in this study, the pressure-viscosity coefficient was not found in the literature. According to previous studies, the pressure-viscosity coefficient of ILs are generally between 12~21 GPa^{-1} (Pensado et al., 2008; Paredes et al., 2012; Mordukhovich et al., 2013; Gaciño et al., 2015), thus here the film thicknesses of the systems are calculated by assuming the limit values of 12 GPa^{-1} and 21 GPa^{-1} for the pure ILs.

Table S1 shows the calculated film thicknesses do not vary significantly for different chosen values of the pressure-viscosity coefficient.

TABLE S1 | The pressure-viscosity coefficients and calculated film thicknesses h_c for the lubricants used in this study.

Lubricant	h_c (nm) (α = 12 GPa^{-1})		h_c (nm) (α = 21 GPa^{-1})	
	5 N	10 N	5 N	10 N
$P_{6,6,6,14}$ TFSI	18	18	25	24
$P_{8,8,8,6(2)}$ BEHP	55	52	74	70
$P_{6,6,6,14}$ BEHP	40	39	54	52
$P_{6,6,6,14}$ $(^iC_8)_2PO_2$	42	40	56	54
Hexadecane	1	1	1	1

References

Gaciño, F.M., Comuñas, M.J.P., Regueira, T., Segovia, J.J., and Fernández, J. (2015). On the viscosity of two 1-butyl-1-methylpyrrolidinium ionic liquids: Effect of the temperature and pressure. *The Journal of Chemical Thermodynamics* 87**,** 43-51.

Mordukhovich, G., Qu, J., Howe, J.Y., Bair, S., Yu, B., Luo, H., Smolenski, D.J., Blau, P.J., Bunting, B.G., and Dai, S. (2013). A low-viscosity ionic liquid demonstrating superior lubricating performance from mixed to boundary lubrication. *Wear* 301**,** 740-746.

Paredes, X., Fandiño, O., Pensado, A.S., Comuñas, M.J.P., and Fernández, J. (2012). Pressure–Viscosity Coefficients for Polyalkylene Glycol Oils and Other Ester or Ionic Lubricants. *Tribology Letters* 45**,** 89-100.

Pensado, A.S., Comuñas, M.J.P., and Fernández, J. (2008). The Pressure–Viscosity Coefficient of Several Ionic Liquids. *Tribology Letters* 31**,** 107-118.

Stachowiak, G.W., and Batchelor, A.W. (2005). *Engineering Tribology.* Elsevier Butterworth-Heinemann.
